# The impact of dyslipidemia on lumbar intervertebral disc degeneration and vertebral endplate modic changes: a cross-sectional study of 1035 citizens in China

**DOI:** 10.1186/s12889-023-16224-3

**Published:** 2023-07-06

**Authors:** Liang Yuan, Zhengqi Huang, Weitao Han, Ruiming Chang, Bo Sun, Mingxi Zhu, Chenjing Li, Jiansen Yan, Bin Liu, Haidong Yin, Wei Ye

**Affiliations:** 1 Department of Spine Surgery, Sun Yat-sen Memorial Hospital, Sun Yat-sen University, Guangzhou, China; 2 Department of Health Examination Center, Sun Yat-sen Memorial Hospital, Sun Yat-sen University, Guangzhou, China; 3 Department of Spine Surgery, The Eighth Affiliated Hospital of Sun Yat-sen University, Shenzhen, China; 4 Department of Spine Surgery, The Third Affiliated Hospital of Sun Yat-sen University, Guangzhou, China; 5 Department of Minimally Invasive Spine Surgery, Panyu Hospital of Chinese Medicine, Guangzhou, China

**Keywords:** Dyslipidemia, Intervertebral disc degeneration, Modic change, Cholesterol, Risk factor

## Abstract

**Background:**

Intervertebral disc degeneration (IDD) and vertebral endplate Modic changes (MCs) are common lumbar degenerative phenotypes related to low back pain (LBP). Dyslipidemia has been linked to LBP but its associations with IDD and MCs have not been fully elucidated. The present study aimed to address the possible link between dyslipidemia, IDD and MCs in the Chinese population.

**Methods:**

1035 citizens were enrolled in the study. The levels of serum total cholesterol (TC), low-density lipoprotein cholesterol (LDL-C), high-density lipoprotein cholesterol (HDL-C) and triglycerides (TG) were collected. IDD was evaluated based on the Pfirrmann grading system and subjects with an average grade ≥ 3 were defined as having degeneration. MCs were classified into typical types 1, 2 and 3. Covariables, including age, sex, BMI and fasting plasma glucose, were included for the adjustment of the logistic analyses.

**Results:**

The degeneration group included 446 subjects while the nondegeneration group included 589 subjects. The degeneration group had significant higher levels of TC and LDL-C (*p* < 0.001) whereas TG and HDL-C were not significantly different between the two groups. TC and LDL-C concentrations were significantly positively correlated with average IDD grades (*p* < 0.001). Multivariate logistic regression revealed that high TC (≥ 6.2 mmol/L, adjusted OR = 1.775, 95% CI = 1.209–2.606) and high LDL-C (≥ 4.1 mmol/L, adjusted OR = 1.818, 95% CI = 1.123–2.943) were independent risk factors for IDD. Type 1 MC presented in 84 (8.12%) subjects, type 2 MC presented in 244 (23.57%) subjects, type 3 MC presented in 27 (2.61%) subjects and no MC was observed in the remaining 680 (65.70%) subjects. The type 2 MC group demonstrated a higher level of TC, but the association between serum lipids and MCs could not be confirmed in further multivariate logistic regression.

**Conclusions:**

High TC (≥ 6.2 mmol/L) and LDL-C (≥ 4.1 mmol/L) concentrations were independent risk factors for IDD for citizens in China. However, the association between dyslipidemia and MCs could not be determined. The effect of excess serum cholesterol may be critical for IDD and cholesterol lowering treatment may provide new opportunities in the management of lumbar disc degeneration.

**Supplementary Information:**

The online version contains supplementary material available at 10.1186/s12889-023-16224-3.

## Introduction

Low back pain (LBP) is a common musculoskeletal complaint across the globe that leads to considerable social and economic burdens [1]. The global burden of disease study has identified LBP as the leading cause of productivity loss and the top cause of years lived with disability [2]. The prevalence of LBP is distributed from teenage years to old ages, even with a potential childhood onset [3]. It is estimated that over 70% of people experience LBP at least once during their lifetime and approximately 40% of the general population continues to suffer chronic LBP [4].

Intervertebral disc degeneration (IDD) is considered one of the main causes of LBP [1]. IDD is an irreversible pathological process characterized by degradation of the extracellular matrix (ECM), dehydration and proteoglycan loss, inflammation, cell senescence and cell death in the nucleus pulposus (NP) [5–7]. The anatomic and biochemical changes during IDD further subject the spine to the loss of mechanical stability and pose threat to adjacent tissues including nerves, ligaments and muscles [8]. Although IDD has been intensively studied in the past two decades, the underlying pathophysiological mechanisms remain incompletely understood.

Modic changes (MCs) are another common lumbar degenerative phenotype that are strongly linked to LBP [9]. MCs are visible vertebral endplate and subchondral bone marrow signal changes observed on magnetic resonance imaging (MRI) scans, and they were first described by Modic et al. in 1988 [10]. Typically, MCs are classified into three types: type 1 MCs represent increased vascularization and edema, type 2 MCs indicate fatty marrow degeneration and type 3 MCs reflect bone sclerosis [9]. It has been proposed that the development of MCs is triggered by mechanical failure and inflammatory response [11]. However, the etiology and potential risk factors for MCs have not yet been clearly elucidated.

Dyslipidemia is a public health epidemic that is of great concern worldwide. The serum lipid level in the Chinese population has gradually increased over the last three decades [12, 13]. According to the China Nutrition Survey, the prevalence of dyslipidemia rose from 18.6% in 2002 to an alarming 40.4% in 2012 [14]. A national survey in 2018 reported that only 39% of Chinese adults had an ideal low-density lipoprotein cholesterol (LDL-C) level (≤ 2.6 mmol/L, as suggested in Chinese guidelines) [15].

It was reported that dyslipidemia has been linked to multiple pathological conditions such as cancers, type 2 diabetes, atherosclerosis, liver disease and osteoarthritis [16]. Recently, the association between abnormal lipid metabolism and LBP has also been revealed [17–19]. In addition, in vivo and in vitro studies have demonstrated that IDD, as the pathological basis of LBP, could be directly exacerbated by excess cholesterol but could also be alleviated by cholesterol-lowering drugs [20–22]. However, epidemiological evidence confirming the relationship between cholesterol and IDD is still lacking. On the other hand, the association between serum lipid levels and MCs has only been explored in the cervical spine, but the results were equivocal [23, 24]. Significant correlations were observed between MCs and TG in the cervical spine [24]. However, whether dyslipidemia plays a role in the development of lumbar spine MCs has not yet been investigated.

The following study addressed the possible link between dyslipidemia, IDD and MCs in citizens from China. We seek to identify risk factors for IDD and MCs among various serum lipid parameters and emphasize the potential epidemiological relation between elevated serum cholesterol and IDD.

## Materials and methods

The ethics committees of Sun Yat-sen Memorial Hospital, Sun Yat-sen University and the ethics committees of the Third Affiliated Hospital, Sun Yat-sen University approved the current study and approved the waiver of written informed consent due to the retrospective nature of the current study. All procedures were conducted in accordance with the Declaration of Helsinki.

### Study population

Data from citizens who underwent an annual health checkup in the aforementioned two hospitals from January 2018 to December 2022 were retrospectively collected. The inclusion criteria were as follows: (1) age over 18 years; (2) complete basic information including age, sex, height and weight; and (3) undertook blood biochemical tests and lumbar magnetic resonance imaging (MRI) scans during the health checkup. The exclusion criteria were as follows: (1) previous history of lumbar surgery; (2) pathological spinal condition, including tuberculosis, tumor, fracture, infection and spine deformity, detectable on an MRI scan; and (3) other major abnormality detected in a health checkup that needed further referral, including secondary causes for dyslipidemia such as hypothyroidism, liver diseases, renal diseases, autoimmune diseases, hematologic diseases [25].

### Serum lipid parameters

Blood samples were collected after an overnight fast that started at 10 p.m. Biochemical analysis was conducted on fresh blood specimens using a fully automated biochemical analyzer following standard procedures (HITACHI 7600 automatic analyzer, Japan; Beckman Coulter AU5821, USA). The results were double-checked by the laboratory physicians. According to the 2016 Chinese guidelines for the management of dyslipidemia in adults [12], a level of total cholesterol (TC) < 5.2 mmol/L was defined as normal, a level between 5.2 mmol/L and 6.2 mmol/L was defined as borderline high, and a level ≥ 6.2 mmol/L was defined as high (hypercholesterolemia). A level of triglycerides (TG) < 1.7 mmol/L was defined as normal, a level between 1.7 mmol/L and 2.3 mmol/L was defined as borderline high, and a level ≥ 2.3 mmol/L was defined as high (hypertriglyceridemia). A level of low-density lipoprotein cholesterol (LDL-C) < 2.6 mmol/L was defined as ideal, a level < 3.4 mmol/L was defined as normal, a level between 3.4 mmol/L and 4.1 mmol/L was defined as borderline high, and a level ≥ 4.1 mmol/L was defined as high. A level of high-density lipoprotein cholesterol (HDL-C) ≥ 1.0 mmol/L was defined as normal, and a level < 1.0 mmol/L was defined as low.

### Assessment of IDD and MCs

Standard lumbar MRI examinations were performed on the 1.5 T Philips Intera or the 3.0 T United Imaging uMR 790. The severity of IDD was assessed in the mid-sagittal T2-weighted image. According to the Pfirrmann classification, lumbar disc degeneration was classified into five grades based on the structure, distinction of nucleus and anulus, signal intensity and disc height [26]. Intervertebral discs (IVDs) from L1/2 to L5/S1 were all graded and the average grade represented the overall severity of IDD. Subjects were assigned to the disk degeneration group if their average grade was ≥ 3 and those with an average grade of < 3 were assigned to the nondegeneration group [27, 28].

MCs were assessed from the caudal endplate of L1 to the cranial endplate of S1. MCs were classified into three categories according to the definition by Modic et al. [10]. Type 1 MC is hypointense signal on T1-weighted images and hyperintense signal on T2-weighted images. Type 2 MC is hyperintense signal on T1-weighted images and hyper- or isointense signal on T2-weighted images. Type 3 MC is hypointense signal on both T1- and T2-weighted images [10]. Subjects with normal vertebral endplate and bone marrow appearances were defined as Type 0 for reference [9]. The current study focused on determining the association between serum lipid metabolism and specific individual types of MCs as different individual type of MCs was related to completely different pathological changes, especially for type 2 MC which represented fatty infiltration [9]. Patients with different types of MCs from L1/2-L5/S1 would prevent that distinction. Therefore, according to previous researches [29–31] and after consulting with experts in medical statistics, patients with different types of MCs were excluded in the current study and for mixed-type MCs, type 1/2 was regrouped to type 1 and type 2/3 was regrouped to type 2.

### Covariables

Age, sex and body mass index (BMI) were included as covariables. Age was measured in years and adjusted in 10-year categories. According to the Chinese classification, BMI < 18.5, 18.5 ≤ BMI < 24.0, 24.0 ≤ BMI < 28.0 and BMI ≥ 28.0 were categorized as underweight, normal weight, overweight and obese, respectively [32]. Fasting plasma glucose (FPG) was included in the current study as a covariable, which could be used to adjust for the potential influence of glucose metabolism disorder on lumbar degeneration. Blood samples were collected and analyzed as mentioned above. FPG ≥ 7.0 mmol/L was classified as diabetes, FPG from 6.1 to 6.9 mmol/L was defined as impaired fasting glucose (IFG) and FPG ≤ 6.0 mmol/L was defined as normal [33]

### Statistical analysis

The interobserver and intraobserver reliabilities for the assessment of IDD and MCs were evaluated by calculating the interclass correlation coefficient (ICC). To determine interobserver reliability, the MRI images of 100 randomly selected subjects were independently measured by two orthopedic surgeons who were blinded to the subjects’ information. After an interval of > 4 weeks, the intraobserver reliability was determined under repeated assessment of the aforementioned 100 subjects.

Descriptive and frequency statistics are generated for all variables. Continuous variables are expressed as the means ± standard deviations (SD) and categorical variables are expressed as percentages. The normality of data distribution and the homogeneity of data variance were verified. Differences between the degeneration and nondegeneration groups and between different types of MCs were assessed with independent sample t tests for continuous variables and with the chi-square test for categorical variables. The correlations between the average grade of disc degeneration and serum lipids were analyzed with Pearson’s correlation coefficients. Logistic regression was conducted to explore the effect of serum lipids on IDD and MCs. Univariate logistic regression of each covariable was first performed to evaluate its impact on IDD and MCs. Covariables that reached a p value of ≤ 0.10 were included in further multivariate logistic regression, and the presence of collinearity was assessed. Covariables were enrolled in the multivariate logistics model stepwise until the fully adjusted model was achieved. Unordered multinomial logistic regression was applied to assess the contribution of serum lipid parameters to different types of MC. The effect of every variable was expressed as an odds ratio (OR) with a 95% confidence interval (CI). All statistical analyses were conducted using IBM SPSS 25 (IBM Corp., Armonk, NY, USA). The critical values for significance were set at* p* < 0.05.

## Results

The baseline characteristics of the subject population are displayed in Table 1. A total of 1035 subjects were enrolled in the current study (493 women and 542 men) with an average age of 49.96 ± 11.06 years old (range: 18–89 yr). The average BMI was 24.69 ± 9.01 kg/m^2^. The percentage of overweight was 38.8% and the percentage of obesity was 10.0%. The overall prevalence of dyslipidemia was 46.0%. The prevalence of hypercholesterolemia (TC ≥ 6.2 mmol/L) was 25.5% and the prevalence of hypertriglyceridemia (TG ≥ 2.3 mmol/L) was 15.2%. For IDD grading, the ICCs for interobserver and intraobserver were 0.919 and 0.937, respectively; for MCs classification, the ICCs for interobserver and intraobserver were 0.902 and 0.925, respectively, which indicated excellent reliability.

**Table 1:** Baseline information of the subjects

Items	N	N%	Mean	SD
Age (yr)			49.96	11.06
Sex				
Female	493	47.6		
Male	542	52.4		
BMI (kg/m^2^)			24.69	9.01
< 18.5	9	0.9		
≥ 18.5 and < 24	521	50.3		
≥ 24 and < 28	402	38.8		
≥ 28	103	10.0		
FPG (mmol/L)			5.55	1.40
≥ 3.9 and < 6.0	857	82.8		
≥ 6.0 and < 6.9	93	9.0		
≥ 7.0	85	8.2		
TC (mmol/L)			5.53	1.12
< 5.2	407	39.3		
≥ 5.2 and < 6.2	364	35.2		
≥ 6.2	264	25.5		
TG (mmol/L)			1.66	1.48
< 1.7	709	68.5		
≥ 1.7 and < 2.3	169	16.3		
≥ 2.3	157	15.2		
LDL-C (mmol/L)			3.48	0.94
< 2.6	169	16.3		
≥ 2.6 and < 3.4	324	31.3		
≥ 3.4 and < 4.1	291	28.1		
≥ 4.1	251	24.3		
HDL-C (mmol/L)			1.44	0.43
< 1.0	159	15.4		
≥ 1.0	876	84.6		

*TC* Total cholesterol, *TG* Triglycerides, *LDL-C* Low-density lipoprotein cholesterol, *HDL-C* High-density lipoprotein cholesterol, *FPG* Fasting plasma glucose, *BMI* Body mass index

The degeneration group included 446 subjects (239 women and 207 men) with an average age of 55.98 ± 9.65 years old. The nondegeneration group included 589 subjects (254 women and 335 men) with an average age of 45.40 ± 9.81 years old. Subjects from the degeneration group had a significantly older age (55.98 ± 9.65 yr vs. 45.40 ± 9.81 yr, *p* < 0.001) whereas BMI was not significantly different between the two groups (25.09 ± 13.30 kg/m2 vs. 24.38 ± 2.93 kg/m2, *p* = 0.207). Regarding serum lipid parameters, subjects from the degeneration group had significantly higher levels of TC (5.68 ± 1.16 mmol/L) and LDL-C (3.65 ± 1.01 mmol/L) than subjects from the nondegeneration group (TC 5.41 ± 1.08 mmol/L and LDL-C 3.38 ± 0.87 mmol/L, *p* < 0.001). A higher level of FPG was also observed in the degeneration group (5.70 ± 1.57 vs. 5.44 ± 1.25, *p* = 0.003). However, no difference was noted in TG and HDL-C between the two groups (Table 2).

**Table 2:** Comparison of basic characteristics between nondegeneration group and degeneration group

Items	Nondegeneration group(*n* = 589)	Degeneration group(*n* = 446)	t value	*p* value
Sex (male/female)	335/254	207/239	-3.353	0.001*
Age (yr)	45.40 ± 9.81	55.98 ± 9.65	-17.314	< 0.001*
BMI (kg/m^2^)	24.38 ± 2.93	25.09 ± 13.30	-1.262	0.207
FPG (mmol/L)	5.44 ± 1.25	5.70 ± 1.57	-2.938	0.003*
TC (mmol/L)	5.41 ± 1.08	5.68 ± 1.16	-3.995	< 0.001*
TG (mmol/L)	1.68 ± 1.62	1.63 ± 1.27	0.606	0.545
LDL-C (mmol/L)	3.38 ± 0.87	3.62 ± 1.01	-4.043	< 0.001*
HDL-C (mmol/L)	1.27 ± 0.30	1.67 ± 6.67	-1.436	0.151

*TC* Total cholesterol, *TG* Triglycerides, *LDL-C* Low-density lipoprotein cholesterol, *HDL-C* High-density lipoprotein cholesterol, *FPG* Fasting plasma glucose, *BMI* Body mass index

^*^p < 0.05

On the other hand, significantly higher prevalence of hypercholesterolemia (31.2% vs. 21.2%, *p* < 0.001) and high LDL-C (30.1% vs. 19.9%, *p* = 0.013) and a significantly lower prevalence of low HDL-C (12.3% vs. 17.7%, *p* = 0.014) were noted in the degeneration group than in the nondegeneration group (Table 3). In the meantime, the proportions of borderline high TC and borderline high LDL-C demonstrated no significant differences between groups. No differences were observed in the incidence of any categories of TG abnormalities (Table 3). The aforementioned results indicated a highly probable association between serum cholesterol levels and the severity of IDD. The incidence of overweight was significantly higher in the nondegeneration group (42.2% vs. 34.2%, *p* = 0.011), the degeneration group demonstrated significantly higher prevalence of diabetes (9.9% vs. 6.8%, *p* = 0.039) and IFG (11.9% vs. 7.0%, *p* = 0.004) (Additional Table 1). A significantly higher prevalence of type 2 (34.3% vs. 14.9%, *p* < 0.001) and type 3 MC (10.5% vs. 2.5%, *p* < 0.001) and a lower incidence of type 1 MC (1.0% vs. 7.1%, *p* < 0.001) were observed in the degeneration group (Additional Table 1).

**Table 3:** Incidence of dyslipidemia between nondegeneration group and degeneration group

Items	Nondegeneration group(n = 589)	Degeneration group(*n* = 446)	*X*^2^	*p* value
TC (mmol/L)				
< 5.2	257(43.6%)	150(33.6%)		
≥ 5.2 and < 6.2	207(35.2%)	157(35.2%)	2.439	0.118
≥ 6.2	125(21.2%)	139(31.2%)	14.336	< 0.001*
TG (mmol/L)				
< 1.7	400(67.9%)	309(69.3%)		
≥ 1.7 and < 2.3	96(16.3%)	73(16.4%)	0.159	0.690
≥ 2.3	93(15.8%)	64(14.3%)	1.051	0.305
LDL-C (mmol/L)				
< 2.6	106(18.0%)	63(14.1%)		
≥ 2.6 and < 3.4	203(34.4%)	121(27.1%)	0.255	0.616
≥ 3.4 and < 4.1	163(27.7%)	128(28.7%)	0.665	0.423
≥ 4.1	117(19.9%)	134(30.1%)	6.579	0.013*
HDL-C (mmol/L)				
< 1.0	104(17.7%)	55(12.3%)	6.038	0.014*
≥ 1.0	485(82.3%)	391(87.7%)		

*TC* Total cholesterol, *TG* Triglycerides, *LDL-C* Low-density lipoprotein cholesterol, *HDL-C* High-density lipoprotein cholesterol

^*^p < 0.05

To further explore the effect of serum lipids on IDD, correlation analysis was first conducted followed by logistic regression. As shown in Table 4, the levels of TC and LDL-C demonstrated a significantly positive correlation with the average grade of IDD (*p* < 0.001). On univariate logistics analysis, older age and female sex (OR = 1.523, 95% CI: 1.189–1.950) were significant risk factors for IDD ((Additional Table 2). An FPG level ≥ 7.0 mmol/L (OR = 1.929, 95% CI: 1.251–2.972) and the presence of type 1 (OR = 1.881, 95% CI: 1.193–2.968) or type 2 (OR = 3.335, 95%CI: 2.458–4.526) MCs were also risk factors for IDD in the univariate logistic analyses (Additional Table 2). However, none of the different categories of BMI were associated with IDD (Additional Table 2). The incidence of overweight was significantly different between groups, and previous studies have reported BMI as an independent risk factor for IDD and dyslipidemia [34–37]. Therefore, from a clinical point of view, BMI was included in the multivariate logistic regression model as an adjusted covariable along with age, sex, MCs and FPG.

**Table 4:** Correlations between the average grade of IDD and serum lipid levels

	TC (mmol/L)	TG (mmol/L)	LDL-C (mmol/L)	HDL-C (mmol/L)
Average grade of IDD	0.111(< 0.001*)	-0.034(0.284)	0.104(< 0.001*)	0.044(0.160)

Correlation coefficients are reported with their *p*-values parentheses

*TC* Total cholesterol, *TG* Triglycerides, *LDL-C* Low-density lipoprotein cholesterol, *HDL-C* High-density lipoprotein cholesterol, *IDD* Intervertebral disc degeneration

^*^p < 0.05

Crude and adjusted ORs and 95% CIs of serum lipid parameters for IDD are displayed in Table 5. Univariate analysis demonstrated that high TC (crude OR = 1.905, 95% CI = 1.391–2.609) and high LDL-C (crude OR = 1.927, 95% CI = 1.294–2.870) were related to increased odds of IDD. These significant associations persisted in the fully adjusted multivariate logistic model that controlled covariables including age, sex, BMI, FPG, MCs and serum lipid parameters, which indicated high TC (adjusted OR = 1.775, 95% CI = 1.209–2.606) and high LDL-C (adjusted OR = 1.818, 95% CI = 1.123–2.943) as independent risk factors for IDD. Although high HDL-C (crude OR = 1.524, 95% CI = 1.071–2.170) was identified as a potential risk factor for IDD in the univariate analysis, no association between high HDL-C and IDD remained after full adjustment with other covariables (adjusted OR = 1.547, 95% CI = 0.973–2.434). On the other hand, an FPG level ≥ 7.0 mmol/L and the presence of type 1 MCs were no longer risk factors for IDD after adjustment, whereas type 2 MCs (OR = 2.546, 95% CI: 1.805–3.593) remained an independent risk factor for IDD (Additional Table 2).

**Table 5:** Multivariate logistic regression of serum lipid parameters for IDD

Items	Crude		Adjusted for age, BMI, sex, FPG, MCs and serum lipid parameters	
	OR	95% CI	OR	95% CI
TC (mmol/L)				
< 5.2	1.00		1.00	
≥ 5.2 and < 6.2	1.299	0.973–1.735	1.336	0.950–1.878
≥ 6.2	1.905*	1.391–2.609	1.775*	1.209–2.606
TG(mmol/L)				
< 1.7	1.00		1.00	
≥ 1.7 and < 2.3	0.984	0.702–1.381	0.728	0.481–1.093
≥ 2.3	0.891	0.627–1.266	0.865	0.557–1.374
LDL-C(mmol/L)				
< 2.6	1.00		1.00	
≥ 2.6 and < 3.4	1.003	0.683–1.473	0.929	0.587–1.468
≥ 3.4 and < 4.1	1.321	0.896–1.948	1.287	0.810–2.042
≥ 4.1	1.927*	1.294–2.870	1.818*	1.123–2.943
HDL-C(mmol/L)				
< 1.0	1.00		1.00	
≥ 1.0	1.524*	1.071–2.170	1.547	0.973–2.434

*TC* Total cholesterol, *TG* Triglycerides, *LDL-C* Low-density lipoprotein cholesterol, *HDL-C* High-density lipoprotein cholesterol, *FPG* Fasting plasma glucose, *BMI* Body mass index, *MCs* Modic changes, *OR* Odds ratio, *CI* Confidence interval

^*^p < 0.05

In the current cohort, type 1 MC presented in 84 (8.12%) subjects, type 2 MC presented in 244 (23.57%) subjects, type 3 MC presented in 27 (2.61%) subjects and no MC was observed in the remaining 680 (65.70%) subjects. Subjects with type 1 (53.37 ± 10.17 yr) and type 2 (54.47 ± 9.66 yr) MCs were significantly older than subjects with no MC (47.99 ± 10.97 yr, *p* < 0.001). A significantly higher level of TC was noted in the type 2 MC group (5.70 ± 1.20 mmol/L) than in the non-MC group (5.48 ± 1.10 mmol/L, *p* = 0.008). A significantly higher level of HDL-C was noted in the type 1 MC group (2.96 ± 15.36 mmol/L) than in the non-MC group (1.31 ± 0.31 mmol/L, *p* = 0.001). The average grade of IDD was significantly higher in the type 1 MC group (2.88 ± 0.65) and type 2 MC group (3.01 ± 0.58) than in the non-MC group (2.68 ± 0.59, *p* = 0.004 and *p* < 0.001, respectively). However, no differences were observed in BMI, FPG, TG and LDL-C between different types of MCs (Table 6).

**Table 6:** Comparison of basic information between different types of MCs

Items	Non-MC group(*n* = 680)	Type 1 MC group(*n *= 84)	Type 2 MC group(*n *= 244)	Type 3 MC group(*n* = 27)	*p* value
Sex (male/female)	364/316	52/32	108/136	18/9	0.007*
Age (yr)	47.99 ± 10.97	53.37 ± 10.17	54.47 ± 9.66	48.22 ± 13.51	< 0.001**
BMI (kg/m^2^)	24.86 ± 10.92	24.37 ± 3.25	24.44 ± 2.74	23.49 ± 2.34	0.798
Average grade of IDD	2.68 ± 0.59	2.88 ± 0.65	3.01 ± 0.58	2.78 ± 0.70	< 0.001***
FPG (mmol/L)	5.48 ± 1.23	5.78 ± 2.22	5.65 ± 1.38	5.83 ± 2.19	0.090
TC (mmol/L)	5.48 ± 1.10	5.52 ± 1.14	5.70 ± 1.20	5.16 ± 0.84	0.019****
TG (mmol/L)	1.61 ± 1.35	1.58 ± 0.95	1.86 ± 1.95	1.41 ± 0.79	0.104
LDL-C (mmol/L)	3.46 ± 0.91	3.42 ± 0.84	3.58 ± 1.04	3.47 ± 1.05	0.353
HDL-C (mmol/L)	1.31 ± 0.31	2.96 ± 15.36	1.32 ± 0.33	1.25 ± 0.32	0.012*****

*MC* Modic change, *TC* Total cholesterol, *TG* Triglycerides, *LDL-C* low-density lipoprotein cholesterol, *HDL-C* High-density lipoprotein cholesterol, *FPG* Fasting plasma glucose, *BMI* Body mass index, *IDD* Intervertebral disc degeneration

^*^p < 0.05

^*^*The age of type 1 and type 2 MC group were significantly older than non-MC group (both *p* < 0.001)

^***^The average grade of IDD was significantly higher in the type 1 MC group and type 2 MC

group as compared with non-MC group (*p* = 0.004 and *p* < 0.001, respectively)

^****^The level of TC was significantly higher in the type 2 MC group as compared with non-MC

group (*p* = 0.008)

^*****^The level of HDL-C was significantly higher in the type 1 MC group as compared with

non-MC group (*p* = 0.001)

Compared with the non-MC group, univariate logistic regression showed that a normal level of LDL-C (crude OR = 2.448, 95% CI: 1.144–5.237) might be related to type 1 MC. On the other hand, univariate logistic regression revealed that high TC (crude OR = 1.467, 95% CI: 1.019–2.112) and borderline high TG (crude OR = 1.603, 95% CI: 1.096–2.346) might be risk factors for type 2 MC. However, after adjustment for age, sex, BMI, IDD and FPG, no associations between serum lipid parameters and any type of MCs could be confirmed, which indicated that serum lipid parameters might not be independent risk factors for MCs (Table 7). For other covariables, IDD (adjusted OR = 2.535, 95% CI: 1.795–3.581) was an independent risk factor for type 2 MC (Additional Table 3).

**Table 7:** Multivariate logistic regression for serum lipid parameters

Types of MCs	Items		Crude OR	95%CI	Adjusted OR	95%CI
Non-MC						
Type 1 MC	TC (mmol/L)	< 5.2	1.00			
		≥ 5.2 and < 6.2	1.045	0.623–1.752	1.495	0.759–2.943
		≥ 6.2	0.917	0.506–1.660	1.732	0.648–4.626
	TG (mmol/L)	< 1.7				
		≥ 1.7 and < 2.3	0.794	0.393–1.605	0.693	0.331–1.450
		≥ 2.3	1.215	0.663–2.228	0.961	0.469–1.969
	LDL-C (mmol/L)	< 2.6				
		≥ 2.6 and < 3.4	2.448*	1.144–5.237	2.375	0.923–5.342
		≥ 3.4 and < 4.1	1.429	0.633–3.222	1.015	0.374–2.756
		≥ 4.1	1.359	0.580–3.181	0.884	0.279–2.799
	HDL-C (mmol/L)	< 1.0				
		≥ 1.0	1.679	0.955–2.953	1.788	0.933–3.426
Type 2 MC	TC (mmol/L)	< 5.2				
		≥ 5.2 and < 6.2	1.119	0.790–1.586	1.117	0.680–1.833
		≥ 6.2	1.467*	1.019–2.112	1.071	0.561–2.046
	TG (mmol/L)	< 1.7				
		≥ 1.7 and < 2.3	1.603*	1.096–2.346	1.452	0.959–2.199
		≥ 2.3	1.258	0.835–1.895	1.222	0.754–1.981
	LDL-C (mmol/L)	< 2.6				
		≥ 2.6 and < 3.4	1.053	0.668–1.660	1.131	0.685–1.866
		≥ 3.4 and < 4.1	1.015	0.639–1.612	0.904	0.478–1.709
		≥ 4.1	1.488	0.942–2.352	1.219	0.599–2.483
	HDL-C (mmol/L)	< 1.0				
		≥ 1.0	1.244	0.834–1.855	1.403	0.881–2.235
Type 3 MC	TC (mmol/L)	< 5.2				
		≥ 5.2 and < 6.2	0.901	0.401–2.022	0.383	0.123–1.193
		≥ 6.2	0.234	0.053–1.044	1.012	0.636–1.357
	TG (mmol/L)	< 1.7				
		≥ 1.7 and < 2.3	1.172	0.430–3.194	1.252	0.436–3.594
		≥ 2.3	0.478	0.110–2.078	0.579	0.122–2.748
	LDL-C (mmol/L)	< 2.6				
		≥ 2.6 and < 3.4	2.899	0.625–13.450	3.066	0.646–14.554
		≥ 3.4 and < 4.1	3.367	0.734–15.453	6.911	0.986–40.797
		≥ 4.1	1.529	0.275–8.485	7.439	0.958–57.788
	HDL-C (mmol/L)	< 1.0				
		≥ 1.0	1.400	0.517–3.785	1.226	0.405–3.710

*TC* Total cholesterol, *TG* Triglycerides, *LDL-C* Low-density lipoprotein cholesterol, *HDL-C* High-density lipoprotein cholesterol, *BMI* Body mass index, *OR* Odds ratio, *CI* Confidence interval, *MC* Modic change

^*^p < 0.05

## Discussion

IDD and MCs are common lumbar degenerative phenotypes closely related to LBP [1, 9]. The current study investigated the impact of abnormal serum lipid metabolism on IDD and MCs from a cohort of 1035 citizens in China. A multivariate logistic regression analysis identified that high TC (≥ 6.2 mmol/L) and high LDL-C (≥ 4.1 mmol/L) were independent risk factors and predictors for IDD. However, no association between serum lipid parameters and any type of MCs was observed.

Previously, several studies have identified that elevated serum lipid levels were related to an increased risk of IDD. In 2008, Hangai et al. [38] analyzed the relationship between cholesterol and the degeneration of every segment disc in a cohort of 270 adults aged over 50 in Japan and revealed that high LDL-C (≥ 3.6 mmol/L) was only correlated with the severity of L4/5 segment disc degeneration. However, in the more recent study of Maurer et al. [37] which included 385 individuals in Germany, there was no significant association between IDD and serum lipids, including HDL-C level, LDL-C level and TG level. Shi et al. [35] reported that in a cohort of 678 individuals in China, only elevated TGs (≥ 1.7 mmol/L) were a significant predictor of disc degeneration. The role of cholesterol in the progression of IDD was not clear until recent in vivo and in vitro experiments demonstrated that excess cholesterol directly aggravated IDD by interfering with the pyroptotic death of NP cells and damaging ECM metabolism [20]. On the other hand, researchers have studied the effect of serum lipids on symptomatic lumbar disc herniation. In the study of Longo et al. [39], patient with symptomatic lumbar disc herniation had significantly higher level of TG and TC concentration compared to controls. Huang et al. [40] also discovered that HDL-C, TG and the ratio of LDL-C/HDL-C were significant contributing factors to symptomatic lumbar disc diseases. The current study was the first to provide direct epidemiological evidence confirming the close relationship between serum cholesterol and IDD, together highlighting the impact of abnormal cholesterol metabolism on IDD.

Currently, the exact pathological mechanism of how serum lipids affect IDD remains largely unclear. Lipid metabolism dysfunction is a well-known manifestation of obesity. However, in the current study, no significant association was noted between BMI and IDD. In another study by Shi et al. [35], patients who were ‘lipid healthy but obese’ and patients who were ‘lipid abnormal but not obese’ also demonstrated different patterns of IDD. Meanwhile, hypercholesterolemia rat models established by high cholesterol diet feeding showed no significant difference in weight gain compared with controls, but the TC and LDL-C levels were significantly elevated and the IDD conditions were significantly accelerated [20]. It was speculated that the altered biomechanics by obesity may impinge on certain segments of the disc strongly while lipid abnormalities may influence IDD systematically [35], highlighting that the effect of serum lipids on overall lumbar spine IDD was more of a biomolecular aspect than a mechanical aspect.

Recently, the biomolecular crosstalk between obesity and IDD has become a particular concern [41]. The condition of overweight and obesity is a systematic inflammatory disorder fueled by continuous and low-grade production of pro-inflammatory cytokines from obese adipose tissue [8]. Adipokines, another proinflammatory protein produced by adipose tissue, have been highlighted as critical regulators in the interaction between metabolic diseases and IDD [41]. The dysregulation of adipokines, such as leptin, adiponectin and visfatin, could also alter the immune microenvironment and affect the homeostasis within the IVD [42–44].

In addition to inflammatory disturbance, excess lipids could also exert a direct deleterious impact on NP cells. Cholesterol is an indispensable composition of cells, but excess cholesterol can damage membrane fluidity, disrupt membrane protein function and hinder signal transduction, which further results in cell dysfunction and death [16]. A recent study discovered that cholesterol accumulated in degenerative NP tissue as IDD developed in both human and hypercholesterolemia rat models, and that exogenous cholesterol could directly interfere with the pyroptotic death of NP cells and damage ECM metabolism [20]. These findings are consistent with the results of the current study, highlighting the direct biomolecular effect of excess serum cholesterol on IDD. Likewise, Wu et al. [45] revealed that oxidized LDL could also induce AF cell apoptosis.

Thus far, the relationship between serum lipid levels and MCs has only been explored in the cervical spine. In a similar cohort of patients with cervical spondylotic myelopathy, Bai et al. [23] revealed that the impact of serum lipids on MCs could not be confirmed but Lv et al. [24] reported significant correlations between MCs and TG. For lumbar MCs, Li et al. [29] used magnetic resonance spectroscopy to analyze the serum metabolomics profiling and revealed that mean diameter of VLDL/LDL particles and cholesterol esters/phospholipid in large LDL were significant metabolomic biomarker for MC and decreased VLDL mean diameter may lead to MCs. However, so far, serum metabolomics profiling and laboratory test for the mean diameter of VLDL/LDL and cholesterol esters/phospholipid in large LDL have not been widely applicable in general clinical practice, which limits their value in predicting and preventing MCs.

The current study first sought to analyze the relationship between serum lipid levels and MCs of the lumbar spine and revealed that subjects with type 2 MCs presented with higher levels of TC than subjects without MCs. Type 2 MCs, which represent yellow fatty marrow infiltrating and replacing the red hemopoietic marrow in histopathology, were considered closely related to lipid metabolism [9]. Bone marrow adiposity of the lumbar vertebral body has also been demonstrated to have significant correlations with serum lipids including TC, TG, HDL and LDL [46, 47]. However, a recent study analyzed the characteristics of 150 endplates with type 2 MCs using an MRI fat suppression sequence but discovered that 75.3% of the type 2 MCs were not suppressed on fat suppression images, suggesting that type 2 MCs may not always represent fat degeneration [48]. The current study was also unable to reveal further association between serum lipids and any type of MCs in the multivariate logistic regression. There is still an urgent need for studies concerning the complex pathological basis of MCs. MCs were considered to represent a dynamic process as the different MC type could convert to each other over time [49]. Further longitudinal studies that focus on the conversion of MCs and fluctuation of serum lipids may provide deeper insight.

It is widely believed that dyslipidemia plays a vital role in the occurrence of atherosclerosis. Intervertebral discs, as the largest avascular tissue in the body, rely on diffusion through the endplate from capillaries in the vertebral bodies to absorb nutrition [50]. Insufficient lumbar blood supply caused by aortic atherosclerosis and stenosis of the lumbar arteries has been associated with an increased risk of IDD [50–53]. On the other hand, the vertebral endplate could also be viewed as an end organ of vascular supply [11]. The associations between MCs and cardiovascular risk factors such as overweight/obesity and smoking have been established in previous studies [54, 55].

The nucleus pulposus, annulus fibrosus, cartilage endplate and subchondral bone marrow are adjacent structures that actively and continuously interact with each other in biomechanical and biochemical aspects, especially during the development of IDD and MCs. The status of the disc’s integrity was associated with the presence of MCs [55]. The loss of normal nucleus pulposus structure would generate excessive biomechanical stresses that could be transmitted to the cartilage endplate and result in cartilage endplate microdefects [54, 56]. Structural defects in the cartilage endplate creates channel for biochemical mediators form the degenerative nucleus pulposus to stimulate inflammation and tissue degradation in the cartilage endplate and subchondral bone marrow [54, 56].

On the other hand, an intact cartilage endplate is also an effective biological barrier that protects the nucleus pulposus from the circulating inflammatory mediators; such obstructive effect would be compromised during MCs, allowing transport of these mediators and promoting IDD [23, 57]. Meanwhile, cartilage endplate defects would also affect its permeability, which hinder the nutrient diffusion and the removal of metabolic waste of the nucleus pulposus [58, 59]. Although IDD and MCs were closely related, the current study discovered that serum lipid metabolism was associated with IDD but no MCs. The different pathological basis between IDD and MCs, as well as the different metabolic characteristics, tissue structure, blood supply and biological function between nucleus pulposus, annulus fibrosus, cartilage endplate and subchondral bone marrow, may explain this discrepancy under dyslipidemia.

Since the associations between abnormal lipid metabolism and lumbar degeneration were established, the potential of lipid-lowering drugs in the treatment and prevention of IDD has been brought into focus. A clinical study elucidated that a higher dosage of statin administration could lower the risk of spine degeneration in patients with hypercholesterolemia [60]. A previous study demonstrated that the IDD condition of hypercholesterolemia rat models could be ameliorated by the administration of atorvastatin by decreasing serum cholesterol levels [20]. The anti-inflammatory aspect of statins could also present protective effects during IDD. Recent studies identified that atorvastatin and rosuvastatin could abolish the TNF-α-induced catabolic effect on NP cells by inhibiting inflammation-related activities in vivo and in vitro [21, 22]. Given the aforementioned epidemiological, clinical and experimental evidence, it is reasonable to extrapolate that hypolipidemic treatment, especially when targeting cholesterol, represents new opportunities in the management of lumbar degenerative diseases.

The present study had several strengths. First, the thresholds and categories concerning abnormalities in lipid components changed over time as the epidemiological characteristics of dyslipidemia were altered [13]. The current study applied the latest 2016 Chinese guidelines in defining the different categories of serum lipid abnormalities to provide an up-to-date reference. And the current research focused on the overall lumbar disc degeneration status and provided a specific threshold of TC (≥ 6.2 mmol/L) and LDL-C (≥ 4.1 mmol/L) for predicting citizens with high risk of IDD, which increased the practical value of our findings in promoting the public health status of lumbar degeneration in China. On the other hand, IDD and MCs are well-known significant risk factors for each other [55, 61], but epidemiological studies of this field have not considered such an influence. The logistic regression model of the current study included IDD and MCs as an important adjustment for each other in revealing the actual impact of serum lipid metabolism. FPG was also included in the regression model to adjust for the possible influence of glucose metabolism.

We also acknowledge some limitations to the current study. First, causality could not be confirmed due to the cross-sectional design. Further longitudinal follow-up may reveal a deeper relationship between excessive serum lipids and IDD. Second, the initiation and progression of IDD, MCs and dyslipidemia are multifactorial and complex. The information on the use of lipid-lowering medication was not routinely collected in the general practice of the health checkup. Therefore, the influence of lipid-lowering meditation status could not be ruled out, as well as the influence of other unmeasured covariables, such as lifestyle factors, occupations and comorbidities.

## Conclusions

High TC (≥ 6.2 mmol/L) and high LDL-C (≥ 4.1 mmol/L) concentrations were independent risk factors for IDD in citizens in China. However, the correlation between serum lipid metabolism and MCs could not be confirmed. Elevated serum cholesterol levels are an effective predictor of IDD in the Chinese population. The biomolecular effect of excess serum cholesterol may play a vital role in the development of IDD. For future clinical practice, hypolipidemic treatment, especially when targeting cholesterol, may provide new opportunities in the management of lumbar degenerative diseases.

## Supplementary Information

**Additional file 1:** **Additional Table 1.** Incidence of basiccharacteristics between nondegeneration group and degeneration group. **Additional Table 2.** Univariate logisticanalyses of covariables for IDD. **Additional Table 3.** Multivariate logistic regression of covariablesfor MCs.

## Data Availability

The datasets generated and analyzed during the current study are not publicly available due the fact that they constitute an excerpt of research in progress but are available from the corresponding author on reasonable request.
