# Supplementary material for: The impact of dyslipidemia on lumbar intervertebral disc degeneration and vertebral endplate modic changes: a cross-sectional study of 1035 citizens in China
Source: BMC Public Health. 2023 Jul 6;23:1302. doi: 10.1186/s12889-023-16224-3 (PMC10326938; doi:10.1186/s12889-023-16224-3)
Supplement: Supplementary file 1 — Additional file 1: Additional Table 1. Incidence of basiccharacteristics between nondegeneration group and degeneration group. Additional Table 2. Univariate logisticanalyses of covariables for IDD. Additional Table 3. Multivariate logistic regression of covariablesfor MCs. [file 12889_2023_16224_MOESM1_ESM.docx]

Additional Table 1: incidence of basic characteristics between nondegeneration group and degeneration group

| Items | Nondegeneration group(n=589) | Degeneration group(n=446) | *X*² | P value |
| --- | --- | --- | --- | --- |
| Age (yr) |  |  |  |  |
| <20 | 168(28.5%) | 19(4.3%) |  |  |
| ≥20 and <40 | 384(65.2%) | 256(57.4%) | 58.055 | <0.001* |
| ≥40 | 37(6.0%) | 171(38.3%) | 204.775 | <0.001* |
| BMI (kg/m^2^) |  |  |  |  |
| ≥18.5 and<24 | 279(47.3%) | 242(54.3%) |  |  |
| <18.5 | 5(1.0%) | 4(1.0%) | 0.014 | 0.905 |
| ≥24 and<28 | 249(42.2%) | 153(34.2%) | 6.524 | 0.011* |
| ≥28 | 56(9.5%) | 47(10.5%) | 0.023 | 0.879 |
| FPG (mmol/L) |  |  |  |  |
| ≥3.9 and<6.0 | 508(86.2%) | 349(78.2%) |  |  |
| ≥6.0 and<6.9 | 41(7.0%) | 53(11.9%) | 8.512 | 0.004* |
| ≥7.0 | 40(6.8%) | 44(9.9%) | 4.275 | 0.039* |
| Types of MCs |  |  |  |  |
| Non-MC | 444(75.4%) | 242(54.3%) |  |  |
| 1 | 42(7.1%) | 4(1.0%) | 13.651 | <0.001* |
| 2 | 88(14.9%) | 153(34.3%) | 187.082 | <0.001* |
| 3 | 15(2.5%) | 47(10.5%) | 39.395 | <0.001* |

BMI: body mass index; FPG: fasting plasma glucose; MC: modic change;

**p*<0.05

Additional Table 2: univariate logistic analyses of covariables for IDD

| Items | Crude | | Adjusted | |
| --- | --- | --- | --- | --- |
|  | OR | 95%CI | OR | 95%CI |
| Sex |  |  |  |  |
| Male | 1.00 |  | 1.00 |  |
| Female | 1.523* | 1.189-1.950 | 1.072 | 0.793-1.449 |
| Age (yr) |  |  |  |  |
| <40 | 1.00 |  | 1.00 |  |
| ≥40 and <60 | 5.895* | 3.575-9.719 | 5.156* | 3.082-8.625 |
| ≥60 | 40.865* | 22.591-73.922 | 35.737* | 19.250-66.345 |
| BMI (kg/m^2^) |  |  |  |  |
| <18.5 | 1.00 |  | 1.00 |  |
| ≥18.5 and<24 | 0.922 | 0.245-3.473 | 0.887 | 0.160-4.908 |
| ≥24 and<28 | 1.302 | 0.896-1.948 | 1.264 | 0.808-2.121 |
| ≥28 | 1.876 | 0.974-2.870 | 1.578 | 0.923-2.943 |
| FPG (mmol/L) |  |  |  |  |
| ≥3.9 and<6.0 | 1.00 |  | 1.00 |  |
| ≥6.0 and<6.9 | 1.562 | 0.999-2.442 | 1.242 | 0.737-2.095 |
| ≥7.0 | 1.929* | 1.251-2.972 | 1.326 | 0.795-2.213 |
| Types of MCs |  |  |  |  |
| Non-MC | 1.00 |  | 1.00 |  |
| 1 | 1.881* | 1.193-2.968 | 1.351 | 0.798-2.289 |
| 2 | 3.335* | 2.458-4.526 | 2.546* | 1.805-3.593 |
| 3 | 1.505 | 0.693-3.268 | 1.733 | 0.717-4.189 |

FPG: fasting plasma glucose; BMI: body mass index; MC: modic change;

**p*<0.05

Additional Table 3: multivariate logistic regression of covariables for MCs

| Types of MCs | Items |  | Crude OR | 95%CI | Adjusted OR | 95%CI |
| --- | --- | --- | --- | --- | --- | --- |
| Non-MC | | | | | | |
| Type 1 MC |  |  |  |  |  |  |
|  | Sex | Male | 1.00 |  | 1.00 |  |
|  |  | Female | 0.709 | 0.445-1.129 | 0.673 | 0.404-1.122 |
|  | Age (yr) | <20 | 1.00 |  | 1.00 |  |
|  |  | ≥20 and <40 | 2.425* | 1.126-5.244 | 2.420* | 1.092-5.365 |
|  |  | ≥40 | 4.824* | 2.095-11.106 | 4.135* | 1.627-10.507 |
|  | BMI (kg/m^2^) | ≥18.5 and<24 | 1.00 |  | 1.00 |  |
|  |  | <18.5 | 4.119 | 0.993-17.081 | 4.395 | 0.948-20.370 |
|  |  | ≥24 and<28 | 0.954 | 0.581-1.566 | 0.877 | 0.521-1.475 |
|  |  | ≥28 | 1.075 | 0.500-2.309 | 0.905 | 0.405-2.024 |
|  | FPG (mmol/L) | ≥3.9 and<6.0 | 1.00 |  | 1.00 |  |
|  |  | ≥6.0 and<6.9 | 0.475 | 0.144-1.560 | 0.437 | 0.130-1.472 |
|  |  | ≥7.0 | 1.741 | 0.868-3.492 | 1.393 | 0.665-2.914 |
|  | IDD | Non-degeneration | 1.00 |  | 1.00 |  |
|  |  | Degeneration | 1.881* | 1.193-2.968 | 1.437 | 0.849-2.433 |
| Type 2 MC |  |  |  |  |  |  |
|  | Sex | Male | 1.00 |  | 1.00 |  |
|  |  | Female | 1.451 | 1.081-1.947 | 1.380 | 0.990-1.923 |
|  | Age (yr) | <20 | 1.00 |  | 1.00 |  |
|  |  | ≥20 and <40 | 4.211* | 2.336-7.497 | 2.877* | 1.585-5.222 |
|  |  | ≥40 | 8.270* | 4.439-15.406 | 3.715* | 1.881-7.335 |
|  | BMI (kg/m^2^) | ≥18.5 and<24 | 1.00 |  | 1.00 |  |
|  |  | <18.5 | 1.245 | 0.932-1.512 | 1.324 | 0.917-5.361 |
|  |  | ≥24 and<28 | 1.155 | 0.847-1.575 | 1.238 | 0.887-1.727 |
|  |  | ≥28 | 1.020 | 0.613-1.697 | 0.904 | 0.525-1.558 |
|  | FPG (mmol/L) | ≥3.9 and<6.0 | 1.00 |  | 1.00 |  |
|  |  | ≥6.0 and<6.9 | 1.641* | 1.007-2.673 | 1.306 | 0.777-2.194 |
|  |  | ≥7.0 | 1.582 | 0.966-2.591 | 1.143 | 0.675-1.935 |
|  | IDD | Non-degeneration |  |  | 1.00 |  |
|  |  | Degeneration | 3.335* | 2.458-4.526 | 2.535* | 1.795-3.581 |
| Type 3 MC |  |  |  |  |  |  |
|  | Sex | Male | 1.00 |  | 1.00 |  |
|  |  | Female | 0.576 | 0.255-1.300 | 0.682 | 0.284-1.639 |
|  | Age (yr) | <20 | 1.00 |  |  |  |
|  |  | ≥20 and <40 | 1.014 | 0.390-2.638 | 0.943 | 0.339-2.627 |
|  |  | ≥40 | 1.286 | 0.663-4.324 | 0.971 | 0.237-3.970 |
|  | BMI (kg/m^2^) | ≥18.5 and<24 | 1.00 |  | 1.00 |  |
|  |  | <18.5 | 0.993 | 0.541-2.709 | 0.984 | 0.389-2.047 |
|  |  | ≥24 and<28 | 0.980 | 0.443-2.618 | 0.897 | 0.392-2.052 |
|  |  | ≥28 | 0.334 | 0.043-2.573 | 0.343 | 0.043-2.743 |
|  | FPG (mmol/L) | ≥3.9 and<6.0 | 1.00 |  | 1.00 |  |
|  |  | ≥6.0 and<6.9 | 1.007 | 0.230-4.402 | 0.993 | 0.218-4.515 |
|  |  | ≥7.0 | 1.510 | 0.437-5.216 | 1.455 | 0.394-5.366 |
|  | IDD | Non-degeneration |  |  |  |  |
|  |  | Degeneration | 1.505 | 0.693-3.268 | 1.726 | 0.705-4.227 |

MC: modic change; TC: total cholesterol; TG: triglycerides; LDL-C: low-density lipoprotein cholesterol; HDL-C: high-density lipoprotein cholesterol; BMI: body mass index; IDD: intervertebral disc degeneration; OR: odds ratio; CI: confidence interval;

**p*<0.05
